# Large Language Models for Psychiatric Diagnosis Based on Multicenter Real-World Clinical Records: Comparative Study

**DOI:** 10.2196/77699

**Published:** 2026-01-13

**Authors:** Maoqian Sun, Jia Yu, Zhuhong Long, Yun Yang, Tao Xiao, Jiaquan Liang, Jun Feng, Huaili Deng, Guoping Huang

**Affiliations:** 1 Mental Health Center, University-Town Hospital of Chongqing Medical University Chongqing China; 2 Sichuan Mental Health Center The Third Hospital of Mianyang Mianyang China; 3 Third People's Hospital of Panzhihua Panzhihua China; 4 The Third People's Hospital of Ganzhou Ganzhou China; 5 The Third People's Hospital of Foshan Foshan China; 6 Lanzhou Third People's Hospital Lanzhou China; 7 Shanxi Provincial Mental Health Center Taiyuan China

**Keywords:** psychiatric disorders, large language models, artificial intelligence, multicenter study, real-world data

## Abstract

**Background:**

Psychiatric disorders are diagnostically challenging and often rely on subjective clinical judgment, particularly in resource-limited settings. Large language models (LLMs) have demonstrated potential in supporting psychiatric diagnosis; however, robust evidence from large-scale, real-world clinical data remains limited.

**Objective:**

This study aimed to evaluate and compare the diagnostic performance of multiple LLMs for psychiatric disorders using multicenter real-world electronic health records (EHRs).

**Methods:**

We retrospectively analyzed 9923 inpatient EHRs collected from 6 psychiatric centers across China, encompassing all ICD-10 (International Statistical Classification of Diseases, Tenth Revision) psychiatric categories. In total, 3 LLMs—GPT-4.0 (OpenAI), GPT-3.5 (OpenAI), and GLM-4-Plus (Zhipu AI)—were evaluated against physician-confirmed discharge diagnoses. Diagnostic performance was assessed using strict accuracy criteria and lenient classification metrics, with subgroup analyses conducted across diagnostic categories and age groups.

**Results:**

GPT-4.0 achieved the highest overall strict diagnostic accuracy (71.7%) and the highest weighted F1-score under lenient evaluation (0.881), particularly for high-prevalence disorders, such as mood disorders and schizophrenia spectrum disorders. Diagnostic performance varied across age groups, with the highest accuracy observed in older adult patients (up to 79.5%) and lower accuracy in adolescents. Across centers, model performance remained stable, with no significant intercenter differences.

**Conclusions:**

LLMs—especially GPT-4.0—demonstrate promising capability in supporting psychiatric diagnosis using real-world EHRs. However, diagnostic performance varies by age group and disorder category. LLMs should be regarded as assistive tools rather than replacements for clinical judgment, and further validation is needed before routine clinical implementation.

## Introduction

Mental disorders are a significant public health concern worldwide; they have high prevalence rates, which are continuously increasing each year [1]. About 20% of the global population is estimated to experience mental illness in their lifetime, placing a substantial burden on both society and health care systems [2]. However, there is an acute scarcity of psychiatric professionals. Many countries have fewer than 5 psychiatrists per 100,000 individuals; this results in delays in providing timely, high-quality diagnostic and therapeutic services for patients with mental health disorders, particularly in resource-limited regions, including China [3]. This medical resource shortage further underscores the urgent need to increase the accuracy and efficiency of psychiatric diagnoses. In addition, psychiatric diagnoses are inherently complex and challenging. Complex etiologies and high symptom heterogeneity characterize mental disorders. The symptom descriptions of patients often depend on subjective expression, with physicians’ medical records and clinical diagnoses also based on their subjective experience [4]. This subjectivity makes diagnoses susceptible to variation among physicians, leading to inconsistent diagnostic outcomes. Therefore, in psychiatry, there is a crucial need to use modern technologies to increase diagnostic efficiency and accuracy [5].

Recently, the widespread adoption of electronic health records (EHRs) has provided a wealth of unstructured data to support the application of artificial intelligence technologies in medical diagnostics, offering new opportunities for diagnosing psychiatric disorders [6]. Large language models (LLMs), including the GPT series, exhibit powerful capabilities in natural language processing and semantic understanding. They can efficiently process unstructured text and have shown great potential in medical diagnostic assistance [7-10].

At present, studies on the application of LLMs in other medical fields have further confirmed their clinical potential. For example, Hasani et al [11] assessed the performance of GPT-4.0 (OpenAI) in standardizing radiology reports; they reported that it generates high-quality standardized radiology reports and significantly improves the efficiency of report generation and data analysis capabilities. Liu et al [12] compared GPT-4.0 with senior neurosurgeons and reported that the performance of GPT-4.0 in complex medical tasks is comparable with that of experienced experts, demonstrating its value in facilitating the diagnosis of complex cases. In addition, Horiuchi et al [13] reported that GPT-4.0 has demonstrated superior diagnostic accuracy in challenging neuroradiology cases, suggesting that generative artificial intelligence technologies can serve as important supplements to physician cognition in complex diagnostic scenarios. Overall, these studies demonstrate the potential application of GPT-4.0 in multidomain diagnostic tasks.

In psychiatry, LLMs have also begun to garner attention. Because psychiatric diagnoses heavily depend on textual descriptions of patient complaints and medical history, LLMs naturally possess significant application advantages in this field. Previous studies have revealed that ChatGPT-4 (OpenAI) can achieve an accuracy rate of 96.1% in the diagnosis of obsessive-compulsive disorder, significantly outperforming that of clinical psychologists (81.5%) and primary care physicians (49.5%) [14]. In 1 study, researchers used the UTH-BERT model and reported that its diagnostic accuracy for medical history in EHRs (74.3%) surpassed that of psychiatrists with intermediate experience (71.5%) [15]. Despite these advances, prior studies on LLM-based psychiatric diagnosis have several limitations. First, the scope of diseases in previous LLM research has often been narrow, with most studies focusing on a single or a small subset of psychiatric disorders (eg, major depressive disorder and obsessive-compulsive disorder) [16]. In contrast, our study simultaneously included all major *ICD-10* (*International Statistical Classification of Diseases, Tenth Revision*) psychiatric categories (F00-F99), thereby providing broader coverage than previous studies. Nevertheless, our analysis was conducted at the level of primary diagnoses within each category, without explicitly addressing comorbidity patterns, which remains an important direction for future research. Second, data sources are often single and lack validation in real-world clinical settings. Current studies rely primarily on simulated cases or data from a single institution [17], making it challenging to capture patient characteristics across diverse regions and health care practices. In the Chinese context, several studies have begun to explore the use of LLMs for psychiatric diagnosis and Chinese-language medical records; however, comprehensive, large-scale validation in real-world psychiatric EHRs—especially with direct comparisons between international and Chinese models—remains limited. Third, the diagnostic performance across age groups has not been thoroughly investigated, with most studies focusing solely on overall accuracy without examining differences in diagnostic performance across age groups (eg, adolescents and older adult patients). Symptoms, disease progression, and treatment plans for psychiatric disorders significantly differ across different age groups. Whether LLMs can adapt to these differences remains unexplored.

To address the aforementioned issues, this study optimized 3 key aspects: data sources, disease coverage, and population segmentation. First, we incorporated real-world EHR data from 6 representative provincial and municipal psychiatric centers across different regions of China. By directly using clinical records to evaluate the model, this approach is better aligned with real-world medical settings, filling the gap left by previous studies that lacked real-world data validation in the Chinese language. Second, we covered all psychiatric disorders listed in the *ICD-10* classification (F0-F9), overcoming the limitations of previous studies, which focused primarily on a single or a few disease types. This design allowed us to evaluate the model’s generalization ability across a broad range of clinical diagnostic tasks. In addition, we conducted fine-grained age stratification, systematically comparing the model’s diagnostic performance across 4 age groups: adolescents, young adults, middle-aged individuals, and older adult patients. This allowed the identification of the model’s adaptation differences across different age groups. In summary, our study, based on large-scale, multicenter real-world data, is the first to comprehensively evaluate the clinical potential of LLMs for psychiatric diagnosis in China.

## Methods

### Research Design and Data Sources

The study data were sourced from 6 provincial and municipal psychiatric centers in China. These centers are located in Sichuan, Gansu, Shanxi, Guangdong, Jiangxi, and other regions, covering Southwest China, Northwest China, North China, South China, and East China, thereby ensuring good regional representation. The dataset included the EHRs of psychiatric inpatients between July 2017 and July 2024. In total, 12,819 cases were initially screened. Among them, 2896 cases were excluded owing to incomplete records or unclear diagnoses. Therefore, 9923 eligible cases were included. The EHR data used in this study consisted exclusively of unstructured narrative text fields, including chief complaints, history of present illness, and psychiatric examination notes. Structured data, such as laboratory test results, medication prescriptions, vital signs, nursing notes, and imaging reports, were not included, as psychiatric diagnosis primarily depends on descriptive symptomatology and mental status examination rather than auxiliary tests. All records included in the model were noted down by attending psychiatrists at the time of admission only. Clinical notes taken during hospitalization were not included, ensuring that the model received only information available at the initial diagnostic encounter and preventing potential temporal information leakage.

### Ethical Considerations

The Ethics Committee of the Sichuan Provincial Mental Health Center approved this study (approval 202460). Considering the retrospective nature of the study, the Ethics Committee exempted the need for informed consent from the patients. All EHR data were fully deidentified in accordance with the Health Insurance Portability and Accountability Act safe harbor method (45 CFR 164.514(b)), with direct identifiers (eg, names, addresses, contact details, and hospital IDs) removed before analysis. In addition, because narrative clinical text can contain residual indirect identifiers, a stratified manual audit of 500 randomly sampled EHR narratives (approximately 5% of the dataset) was conducted to further minimize reidentification risk. Among these 500 records, 7 (1.4%) instances of potential indirect identifiers were identified and corrected before model inference. No direct identifiers were detected in the sampled records.

Model queries were transmitted through encrypted API calls (HTTPS/TLS) rather than public chat interfaces. Although no formal data processing agreement was established with the external provider, the institutional review board explicitly approved the use of deidentified clinical narratives for inference on external LLM servers. It was assured that the procedure complied with local data governance regulations and did not include identifiable personal information. Only deidentified free text—without metadata, patient identifiers, or institutional information—was transmitted, and no protected health information left the institution.

The study adhered to relevant international and domestic privacy frameworks, including General Data Protection Regulation Article 46 for cross-border data transfer, China’s Personal Information Protection Law, and ISO/IEC 27018 guidelines for the protection of personal data in public cloud environments. Nevertheless, we acknowledge that narrative EHR text has inherent deidentification limitations, and we minimized data transmission to mitigate privacy risks.

### Data Preprocessing

To closely replicate real-world clinical settings, the admission diagnosis names were removed from the medical history; no other data were modified, thereby preserving the integrity of the original data [18]. The diagnoses were classified into 11 categories by extracting only the first letter and the 10-digit *ICD-10* codes from Chapter V (Mental and Behavioral Disorders; F0-F9) and other related codes. Specifically, F0 includes organic mental disorders such as dementia; F1 includes substance-related disorders such as alcohol addiction; F2 includes schizophrenia and associated psychotic conditions; F3 includes mood disorders such as depression; F4 includes anxiety and stress-related disorders; F5 includes disorders associated with physiological conditions, such as eating disorders; F6 includes personality and behavioral disorders in adults; F7 includes intellectual disabilities; F8 includes developmental disorders such as autism; and F9 includes behavioral and emotional conditions beginning in childhood, including attention-deficit or hyperactivity disorders [19]. For subgroup analysis, patients were stratified into 4 age groups: adolescents (≤17 years), young adults (18-35 years), middle-aged adults (36-59 years), and older adult individuals (≥60 years). The thresholds at 18 and 60 years follow established conventions in psychiatric and geriatric research [20]. The adult group was further subdivided into young and middle-aged categories to capture differences in onset age, symptom presentation, and comorbidity patterns of major psychiatric disorders across life stages.

### Research Tools and Model Setup

In total, 3 LLMs were selected for evaluation: GPT-4.0 and GPT-3.5 (OpenAI) and GLM-4-Plus (Zhipu AI). GPT-3.5 was chosen as a widely used baseline model because of its broad accessibility and extensive application in previous medical natural language processing tasks, providing a reference point for evaluation [21]. GPT-4.0, a more advanced successor in the GPT series, was included to assess performance improvements driven by enhanced semantic understanding and reasoning capabilities [22]. GLM-4-Plus is one of the most advanced Chinese-optimized LLMs [23]. It was selected to examine the adaptability and potential of domestic LLMs in psychiatric diagnosis using Chinese-language EHRs and to compare their performance with that of international mainstream models under localized application scenarios.

In addition, a unified prompt was used: “请根据以下患者信息给出最可能的诊断。” (“Please provide the most likely diagnosis based on the following patient information”). Because the clinical input consisted of Chinese-language EHR narratives, the instruction prompt was also written in Chinese to maintain monolingual consistency and avoid potential cross-lingual prompting effects. This prompt was intentionally selected to ensure methodological consistency and fair comparability across all LLMs, as varying prompt strategies may introduce model-specific advantages. Zero-shot prompts also reflect a realistic clinical scenario in which clinicians typically expect the model to provide a diagnostic impression directly without additional examples. No English prompt or mixed-language prompt was used at any stage. No prompt engineering or chain-of-thought prompting was applied, as the study aimed to evaluate each model’s baseline diagnostic capability under identical input conditions. The preprocessed data included the patient’s sex, age, chief complaints, medical history, and psychiatric examination results. The psychiatric examination results included narrative descriptions of key mental status domains, such as appearance and behavior, mood and affect, thought process and content, cognitive function, and insight, which were documented in a standardized format across all participating centers to ensure structural consistency and data comparability. These inputs were provided to the model to generate diagnostic results. All diagnostic analyses by the LLMs were completed between January 1 and January 31, 2025. The discharge diagnosis served as the reference standard and was confirmed by a senior associate chief physician with over 10 years of clinical experience.

Model outputs were evaluated using a 0-2 point scoring system: 2 points for a completely correct diagnosis (exact *ICD-10* match), 1 point for a partially correct diagnosis, and 0 points for an incorrect diagnosis. “Partially correct” was operationally defined as cases in which the predicted diagnosis was clinically close to, but not an exact, *ICD-10* match. Examples included (1) misclassification within the schizophrenia spectrum (eg, schizotypal disorder scored as schizophrenia), (2) assigning a broader category within mood disorders without specifying the exact episode (eg, manic episode scored as mood disorder), or (3) capturing the correct diagnostic domain at a broader level of granularity (eg, generalized anxiety disorder scored as anxiety disorder). Predictions that did not meet these criteria were considered incorrect.

To ensure reliability and minimize bias, multiple 2-rater groups were formed, each comprising 2 attending psychiatrists with more than 5 years of independent clinical experience. Both raters independently scored each case after the model outputs were anonymized and randomized, which blinded them to the source model. Disagreements were resolved by consensus with a senior psychiatrist. Interrater reliability was assessed using Cohen κ coefficient, which demonstrated substantial agreement (Cohen κ=0.82).

### Data Analysis

Statistical analyses were conducted using SPSS Statistics (version 27.0; IBM Corp). Descriptive statistics were used to present the demographic characteristics and distributions of disease categories. Model performance was evaluated by comparing the predicted diagnoses with the discharge diagnoses, which served as the reference standard. Accuracy was calculated as the proportion of correct predictions (score=2). In this study, strict evaluation criteria were used as the primary analytic framework. Under this explicit definition, only exact matches between the model-predicted *ICD-10* diagnosis and the discharge diagnosis (score=2) were considered correct, whereas partially correct predictions (score=1) were treated as incorrect. For the classification performance metrics—namely, precision, recall, and *F*_1_-score—we also applied a lenient evaluation. Under this lenient definition, both exact matches (score=2) and partially correct predictions (score=1) were treated as correct and counted as true positives (TPs). In this framework, TPs were predictions that were exactly or partially consistent with the true diagnosis (score=2 or score=1); false positives (FPs) were predictions for a class when the true diagnosis belonged to another class; and false negatives (FNs) were cases in which the true diagnosis belonged to a class, but the model prediction did not. Predictions with score=0 were always treated as incorrect. These lenient metrics were used exclusively for the classification performance results and are clearly labeled as such. All other analyses throughout the manuscript relied solely on the strict evaluation criteria. In addition, performance metrics, including precision, recall, and *F*_1_-score, were computed under a strict definition: only exact matches between the model-predicted *ICD-10* diagnosis and the discharge diagnosis were considered correct. In this framework, TPs are cases where the prediction exactly matches the true diagnosis; FPs are predictions for a class when the true diagnosis belongs to another class; and FNs are cases where the true diagnosis belongs to a class, but the model prediction does not. Partially correct outputs (score=1) were treated as incorrect predictions and classified as FPs or FNs depending on the prediction-reference mismatch; only score=2 outputs were counted as TPs.

The macro- and weighted-average *F*_1_-scores were calculated to measure overall performance. Because the F8 category contained only 8 cases (0.1% of the dataset), macroaverage *F*_1_-scores were additionally reported, both including and excluding this extremely rare category, to provide a more stable estimate of overall performance. Tests of normality and homogeneity of variance were conducted. The Shapiro-Wilk test indicated that the score data significantly deviated from normality across all model groups, and the Levene test confirmed unequal variances (Table S1 in Multimedia Appendix 1). Therefore, nonparametric rank-sum tests were applied for intergroup comparisons. In addition, Kruskal-Wallis tests were used to examine whether diagnostic accuracy differed across the 6 centers for each model (Table S2 in Multimedia Appendix 1). A *P* value of <.05 indicated significance. To further compare diagnostic performance across LLMs, an ordinal logistic regression analysis was conducted. Because the diagnostic outcome was an ordered 0-2 score (0=incorrect, 1=partially correct, and 2=correct), a proportional odds model was applied to estimate the likelihood of achieving a higher diagnostic score for each model. GPT-3.5 served as the reference category. Odds ratios (ORs) and 95% CIs were calculated to quantify differences in diagnostic performance. The results of this analysis are presented in Table S3 in Multimedia Appendix 1.

## Results

### Demographic Characteristics and Disease Category Distribution

In total, 9923 patients (3665, 36.93% males and 6258, 63.07% females) were included in this study. Across age groups, 1881 (18.96%) were adolescents (≤17 years), 2468 (24.87%) were young adults (18-35 years), 3695 (37.24%) were middle-aged adults (36-59 years), and 1879 (18.94%) were older adults (≥60 years). The disease categories were distributed as follows (Table 1): mood disorders (F3) were the most common, accounting for 36.7% (3646/9923) of the cases, followed by schizophrenia, schizotypal, and delusional disorders (F2, 2470/9923, 24.9%), and neurotic disorders (F4, 1991/9923, 20.1%). The distribution of other disorders was relatively low.

**Table 1:** Demographic data and clinical characteristics.

Characteristics		Value
Sex, n (%)		
	Male	3665 (36.93)
	Female	6258 (63.07)
Age (y), mean (SD)		40.5 (20.03)
Age category, n (%)		
	Adolescent (≤17 y)	1881 (19)
	Young adult (18-35 y)	2468 (24.9)
	Middle-aged (36-59 y)	3695 (37.2)
	Older adult (≥60 y)	1879 (18.9)
Disorder category, n (%)		
	F0 (Organic disorders)	597 (6)
	F1 (Substance use disorders)	243 (2)
	F2 (Schizophrenia, schizotypal, and delusional disorders)	2470 (24.9)
	F3 (Mood disorders)	3646 (36.7)
	F4 (Neurotic disorders)	1991 (20.1)
	F5 (Eating disorders, etc)	70 (0.7)
	F6 (Personality disorders)	86 (0.9)
	F7 (Mental retardation)	177 (1.8)
	F8 (Autism, etc)	8 (0.1)
	F9 (Behavioral disorders)	635 (6.4)

### Diagnostic Accuracy of the 3 Models

**Figure 1:**
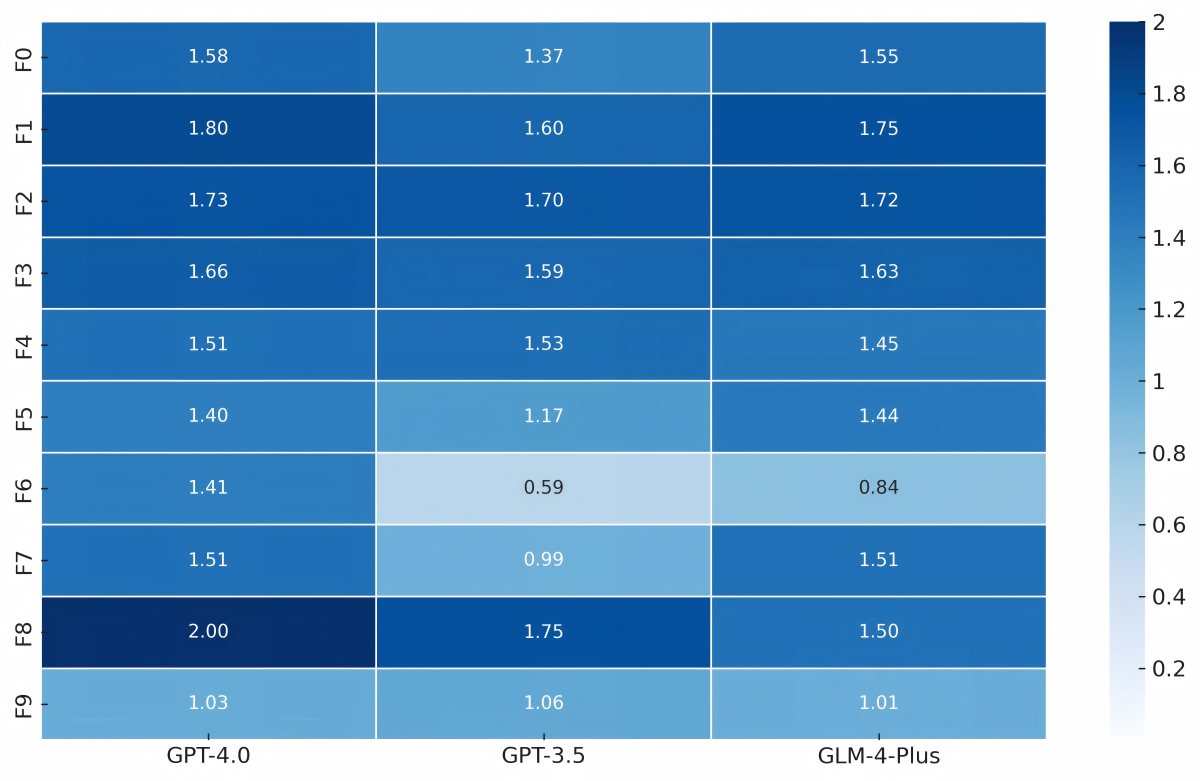
Heatmap of the diagnostic scores. The color intensity indicates the model’s diagnostic score (on a 2-point scale), with darker colors indicating higher scores. This reflects the diagnostic accuracy of the models in each category.

Overall, GPT-4.0 achieved an overall accuracy of 71.7%, outperforming GLM-4-Plus (69.3%) and GPT-3.5 (68.8%). In terms of category-specific performance, GPT-4.0 exhibited higher overall accuracy than the other models in the F1, F3, and F6 categories. However, in the F2 and F4 categories, the diagnostic accuracy rates of the 3 models were similar, with no significant differences. Among the different age groups, diagnostic accuracy was higher in the older adult group (with an overall accuracy rate of 74.3%-79.5%). Additional analysis using the rank-sum test revealed significant differences between adolescents and middle-aged adults, adolescents and older adults, young adults and middle-aged adults, and young adults and older adults (*P*<.001). The results are summarized in Tables 2 and 3, and the category-level diagnostic score patterns across the 3 models are visualized in Figure 1.

**Table 2:** Diagnostic accuracy.

Category		GPT-4.0, n (%)				GPT-3.5, n (%)				GLM-4-Plus, n (%)		
		Correct	Partially correct	Incorrect	Correct		Partially correct	Incorrect	Correct		Partially correct	Incorrect
Disorder category												
	F0	394 (66)	152 (25.5)	51 (8.5)	338 (56.6)		143 (24)	116 (19.4)	415 (69.5)		94 (15.7)	88 (14.7)
	F1	208 (85.6)	22 (9.1)	13 (5.3)	182 (74.9)		25 (10.3)	36 (14.8)	206 (84.8)		14 (5.8)	23 (9.5)
	F2	1906 (77.2)	460 (18.6)	104 (4.2)	1834 (74.3)		531 (21.5)	105 (4.3)	1890 (76.5)		475 (19.2)	105 (4.3)
	F3	2738 (75.1)	561 (15.4)	347 (9.5)	2654 (72.8)		489 (13.4)	503 (13.8)	2576 (70.7)		773 (21.2)	297 (8.1)
	F4	1347 (67.6)	310 (15.6)	334 (16.8)	1368 (68.7)		308 (15.5)	315 (15.8)	1281 (64.3)		323 (16.2)	387 (19.4)
	F5	48 (68.6)	2 (2.9)	20 (28.6)	41 (58.6)		0 (0)	29 (41.4)	50 (71.4)		1 (1.4)	19 (27.1)
	F6	59 (68.6)	3 (3.5)	24 (27.9)	20 (23.3)		11 (12.8)	55 (64)	36 (41.9)		0 (0)	50 (58.1)
	F7	123 (69.5)	22 (12.4)	32 (18.1)	83 (46.9)		9 (5.1)	85 (48)	131 (74)		6 (3.4)	40 (22.6)
	F8	8 (100)	0 (0)	0 (0)	7 (87.5)		0 (0)	1 (12.5)	6 (75)		0 (0)	2 (25)
	F9	285 (44.9)	85 (13.4)	265 (41.7)	296 (46.6)		78 (12.3)	261 (41.1)	282 (44.4)		79 (12.4)	274 (43.1)
Age category												
	Adolescent (≤17 y)	1148 (61)	367 (19.5)	366 (19.5)	1116 (59.3)		341 (18.1)	424 (22.5)	1010 (53.7)		497 (26.4)	374 (19.9)
	Young adult (18-35 y)	1727 (70)	480 (19.4)	261 (10.6)	1638 (66.4)		456 (18.5)	374 (15.2)	1654 (67)		534 (21.6)	280 (11.3)
	Middle-aged (36-59 y)	2759 (74.7)	530 (14.3)	406 (11)	2672 (72.3)		551 (14.9)	472 (12.8)	2715 (73.5)		540 (14.6)	440 (11.9)
	Older adult (≥60 y)	1482 (78.9)	240 (12.8)	157 (8.4)	1397 (74.3)		246 (13.1)	236 (12.6)	1494 (79.5)		194 (10.3)	191 (10.2)
Total		7116 (71.7)	1617 (16.3)	1190 (12)	6823 (68.8)		1594 (16.1)	1506 (15.2)	6873 (69.3)		1762 (17.8)	1285 (12.9)

**Table 3:** Comparison of diagnostic scores based on age.

Age category	*P* value		
	GPT-4.0	GPT-3.5	GLM-4-Plus
Adolescent^a^ vs young adult^b^	<.001	<.001	<.001
Adolescent vs middle-aged^c^	<.001	<.001	<.001
Adolescent vs older adult^d^	<.001	<.001	<.001
Young adult vs middle-aged	<.001	<.001	<.001
Young adult vs older adult	<.001	<.001	<.001
Middle-aged vs older adult	<.001	.15	<.001

^a^Adolescent: ≤17 years.

^b^Young adult: 18-35 years.

^c^Middle-aged: 36-59 years.

^d^Older adult: ≥60 years.

### Center-Level Diagnostic Performance

To evaluate whether model performance varied across institutions, a supplementary center-level analysis was conducted. The diagnostic accuracies of all 3 models demonstrated only minor numerical fluctuations across the 6 centers. Importantly, the Kruskal-Wallis test indicated no statistically significant differences for any of the models (GPT-4.0: *P*=.52; GPT-3.5: *P*=.47; and GLM-4-Plus: *P*=.61). The detailed results for each center are provided in Table S2 in Multimedia Appendix 1.

### Analysis of Differences in Scoring Distribution of 3 Models

The rank-sum test was used to compare differences in the scoring distributions of GPT-4.0, GPT-3.5, and GLM-4-Plus across various *ICD-10* categories. We observed that GPT-4.0 had higher scores than GPT-3.5 in the F0 (*P*<.001), F6 (*P*<.001), and F7 (*P*<.001) categories. Furthermore, GLM-4-Plus exhibited differences in its scoring distribution compared with GPT-3.5 in the F0 (*P*<.001) and F7 (*P*<.001) categories. Overall, the score distribution of GPT-4.0 was superior to that of both GPT-3.5 (*P*<.001) and GLM-4-Plus (*P*<.001). However, the difference in the scoring distribution between GPT-3.5 and GLM-4-Plus was not statistically significant (*P*=.09). Table 4 summarizes the results.

**Table 4:** Comparison of the diagnostic scores of the different large language models.

Disorder category	*P* value		
	GPT-4.0 vs GPT-3.5	GPT-4.0 vs GLM-4-Plus	GPT-3.5 vs GLM-4-Plus
F0	<.001	.69	<.001
F1	.002	.67	.008
F2	.02	.60	.08
F3	.002	<.001	.77
F4	.43	.02	.002
F5	.17	.75	.098
F6	<.001	<.001	.14
F7	<.001	.63	<.001
F8	.38	.17	.59
F9	.65	.72	.42
Total	<.001	<.001	.09

### Comparison of the Classification Performance of 3 Models

Table 5 summarizes the precision, recall, and *F*_1_-scores of the GPT-4.0, GPT-3.5, and GLM-4-Plus models across various *ICD-10* disease categories. To enhance interpretability, these classification metrics were computed using a lenient evaluation framework, in which both exact matches (score=2) and partially correct predictions (score=1) were treated as correct. GPT-4.0 achieved the highest *F*_1_-scores in the F0 (0.915), F1 (0.926), and F2 (0.929) categories. GLM-4-Plus achieved the highest precision in the F2 category (0.951), whereas GPT-4.0 had a superior recall rate (0.959). Small-sample categories exhibited significant fluctuations, including the F8 category, where GPT-4.0’s recall rate reached 1.00, while its precision was only 0.308. This phenomenon reflects class imbalance and overprediction in rare categories. The macroaverage *F*_1_-scores (including F8) were 0.655, 0.410, and 0.712 for GPT-4.0, GPT-3.5, and GLM-4-Plus, respectively. The weighted average *F*_1_-score of GPT-4.0 (0.881) was superior to that of both GPT-3.5 (0.849) and GLM-4-Plus (0.873). To mitigate the instability caused by the extremely small F8 category, macroaverage *F*_1_-scores were also calculated after excluding F8. The exclusion yielded more stable macrolevel estimates: 0.821 for GPT-4.0, 0.745 for GPT-3.5, and 0.806 for GLM-4-Plus.

To further complement this evaluation, we also performed an ordinal logistic regression analysis using the 0-2 scoring system (0=incorrect, 1=partially correct, and 2=correct) as the outcome variable, accounting for its ordinal nature. The results were consistent with the overall performance patterns observed in the main analysis. GPT-4.0 was significantly more likely to achieve a higher diagnostic score compared with GPT-3.5 (OR 1.18, 95% CI 1.11-1.25; *P*<.001). In contrast, GLM-4-Plus showed slightly lower odds of achieving a higher score relative to GPT-3.5 (OR 0.95, 95% CI 0.90-1.01). Although this difference did not reach statistical significance (*P*=.09; Table S3 in Multimedia Appendix 1).

**Table 5:** Precision, recall, and F1-scores of the different large language models (lenient evaluation).

Category^a^			GPT-4.0							GPT-3.5							GLM-4-Plus				
			Precision		Recall		*F*_1_-score		Precision			Recall		*F*_1_-score		Precision			Recall		*F*_1_-score
Disorder category																					
	F0	0.916		0.915		0.915		0.897			0.814		0.853		0.857			0.929		0.891	
	F1	0.906		0.947		0.926		0.924			0.852		0.887		0.880			0.961		0.919	
	F2	0.902		0.959		0.929		0.850			0.961		0.902		0.951			0.876		0.912	
	F3	0.857		0.906		0.881		0.849			0.864		0.856		0.884			0.862		0.873	
	F4	0.913		0.835		0.872		0.859			0.843		0.851		0.863			0.867		0.865	
	F5	0.549		0.714		0.621		0.519			0.586		0.550		0.785			0.607		0.685	
	F6	0.554		0.721		0.626		0.405			0.372		0.388		0.750			0.409		0.529	
	F7	0.973		0.819		0.890		0.979			0.525		0.684		0.820			0.913		0.864	
	F8	0.308		1.000		0.471		0.159			0.875		0.269		0.667			0.500		0.571	
	F9	0.962		0.592		0.733		0.971			0.589		0.733		0.576			0.960		0.720	
Macro average			0.653		0.701		0.655		0.436			0.428		0.410		0.730			0.717		0.712
Macro average (excluding F8)			—^b^		—		0.821		—			—		0.745		—			—		0.806
Weighted average			0.888		0.882		0.881		0.859			0.851		0.849		0.882			0.871		0.873

^a^A lenient evaluation framework was used for all metrics in this table. Both score=2 (exact match) and score=1 (partially correct) predictions were considered as correct.

^b^Not applicable.

## Discussion

### Principal Findings

This multicenter real-world study is the first to systematically evaluate the diagnostic performance of 3 LLMs—GPT-4.0, GPT-3.5, and GLM-4-Plus—across the full spectrum of *ICD-10* psychiatric categories via Chinese psychiatric EHRs. The results demonstrate that GPT-4.0 achieved the highest overall strict diagnostic accuracy (71.7%), followed by GLM-4-Plus (69.3%) and GPT-3.5 (68.8%), showing a clear performance gradient across model generations. In addition to strict accuracy, GPT-4.0 also achieved a higher lenient *F*_1_-score (0.881), indicating performance when partially correct predictions are counted as correct. These complementary metrics highlight different aspects of diagnostic ability under distinct evaluation frameworks.

Consistent with these findings, the ordinal logistic regression provided a quantitative estimate of these differences: GPT-4.0 was significantly more likely to achieve a higher diagnostic score compared with GPT-3.5 (OR 1.18, 95% CI 1.11-1.25; *P*<.001). In contrast, GLM-4-Plus demonstrated ORs comparable with those of GPT-3.5 (OR 0.95, 95% CI 0.90-1.01), indicating no statistically significant difference in performance between the 2 models. These results further support the interpretation that GPT-4.0 provides a measurable performance advantage, whereas GLM-4-Plus and GPT-3.5 exhibit broadly similar diagnostic capability.

Significant differences were observed across most age groups, with diagnostic accuracy generally increasing with age. Older adult patients exhibited notably better diagnostic performance than adolescents and young adults. For GPT-4.0 and GLM-4-Plus, all pairwise adjacent age-group comparisons were statistically significant (all *P*<.001). For GPT-3.5, accuracy also increased with age overall; however, the comparison between middle-aged and older adult patients did not reach statistical significance (*P*=.15). This trend likely reflects several clinical and documentation-related factors: older adults typically present with more stable symptom trajectories, longer disease histories, and more structured and complete EHR documentation, whereas younger patients often exhibit heterogeneous and fluctuating emotional and behavioral symptoms, which increase diagnostic uncertainty.

Across diagnostic categories, GPT-4.0 consistently showed stronger contextual reasoning ability and more accurate extraction of relevant diagnostic cues. It performed particularly well in mood disorders (F3), personality disorders (F6), and several neurotic and stress-related disorders (F4). GLM-4-Plus demonstrated strong performance in schizophrenia spectrum disorders (F2), suggesting an advantage in identifying disorders with clearly structured symptom patterns. GPT-3.5 exhibited the highest rate of misclassification, particularly between disorders with overlapping emotional or behavioral symptoms.

In low-prevalence categories, such as pervasive developmental disorders (F8), all 3 models showed instability in performance. GPT-4.0 achieved perfect recall but exhibited low precision, suggesting overidentification in categories with limited diagnostic cues. These patterns highlight the ongoing challenge for general-purpose LLMs in identifying rare psychiatric conditions.

Beyond model comparison, this study demonstrates the overall feasibility of applying large-scale language models to real-world psychiatric EHRs covering all *ICD-10* categories. The results indicate that GPT-4.0 can extract diagnostically relevant information efficiently and generate multidimensional diagnostic suggestions, thereby supporting initial clinical screening, reducing physician workload, and improving efficiency in high-volume psychiatric settings. However, LLM-based diagnosis remains dependent on the quality of EHRs, symptom clarity, and physician interpretation, and cannot be used as a substitute for clinical expertise or individualized assessment.

Overall, GPT-4.0 demonstrated the strongest, most stable, and most clinically meaningful diagnostic performance across age groups and psychiatric categories, supporting its potential value as a diagnostic assistance tool within real-world psychiatric workflows.

### Center-Level Consistency and Its Implications

Although the 6 centers differed in documentation habits, patient demographics, and narrative styles, all 3 LLMs demonstrated stable diagnostic performance across sites. This consistency suggests that the models rely primarily on core clinical symptom patterns rather than center-specific narrative conventions, which may explain their robustness in real-world settings. The absence of center-level variability also indicates that LLM-based diagnostic support may generalize well across institutions with heterogeneous clinical workflows—a critical requirement for real-world deployment. From an implementation perspective, these findings imply that LLM-assisted diagnostic tools may not require site-specific retraining or local optimization, substantially reducing the technical and operational burden associated with clinical adoption. Collectively, these observations highlight the scalability and practical utility of LLMs in psychiatric diagnostic workflows.

### Misclassification Patterns in the F8 Category

The extreme imbalance between recall and precision in the F8 category warrants further clinical interpretation. Although GPT-4.0 successfully identified all true F8 cases, its low precision indicates a substantial number of FP predictions. Upon reviewing these cases, we found that most FPs originated from F9 (behavioral and attention-deficit/hyperactivity disorder–related disorders), with a smaller proportion from F7 (intellectual disabilities). These diagnostic groups share overlapping behavioral and neurodevelopmental characteristics with F8—including impulsivity, irritability, social dysfunction, and developmental impairment—which may lead the model to rely on broad behavioral descriptors when more specific differentiating features are not explicitly documented in the narrative EHR. This overdiagnosis pattern suggests that GPT-4.0 is sensitive to general behavioral dysregulation but is less effective in distinguishing between rare neurodevelopmental disorders with partially overlapping phenotypes. Understanding this tendency toward misclassification is essential for evaluating the model’s safety profile when applied to clinically rare categories.

### Comparison With Previous Work

The superior diagnostic performance of GPT-4.0 observed in this study is consistent with previous evaluations, which report that newer-generation LLMs outperform earlier versions in clinical reasoning tasks [24-26]. This performance advantage has been attributed to the enhanced ability of GPT-4.0 to capture semantic associations and long-range contextual patterns in complex textual inputs [27]. Previous studies have demonstrated that GPT-4.0 provides higher diagnostic accuracy, better contextual understanding, and more stable reasoning patterns than does GPT-3.5 across diverse clinical applications. Our findings extend this body of evidence to the domain of psychiatric diagnosis via Chinese-language EHRs in real-world settings.

A major distinction between our study and earlier work is the use of multicenter real-world psychiatric EHR narratives rather than simulated vignettes or single-institution datasets. The complexity and variability of real-world clinical documentation allow for a more realistic assessment of LLM behavior in routine psychiatric practice, thereby providing stronger ecological validity.

Consistent with the broader literature, earlier-generation models, such as the GPT-3.5, tend to perform less reliably in disorders characterized by overlapping emotional or behavioral symptoms [24-26]. In our study, this general pattern was reflected in GPT-3.5’s tendency to misclassify personality disorders (F6) as mood disorders (F3), likely due to the similarity and ambiguity of symptom descriptions in these categories within real-world psychiatric EHRs. This observation aligns with the known challenges faced by earlier LLMs in distinguishing clinically overlapping symptom domains. In contrast, GLM-4-Plus demonstrated strong performance in schizophrenia spectrum disorders (F2), suggesting that models trained on Chinese corpus characteristics may perform well in categories with clear and structured symptom patterns.

Beyond diagnostic accuracy, prior studies have highlighted the potential role of LLMs as clinical decision support tools capable of improving workflow efficiency and reducing clinician workload in real-world health care settings [28]. In psychiatry, recent evaluations have summarized the diagnostic accuracy of LLMs and emphasized their potential utility in assisting clinicians with complex diagnostic decisions [29]. More broadly, advances in deep learning–based electronic health record analytics have demonstrated the feasibility of extracting clinically meaningful information from narrative EHRs to support timely screening and downstream decision-making processes [30].

Taken together, our findings reinforce the performance advantages of GPT-4.0 observed in previous work and highlight the importance of evaluating both international and domestic LLMs in localized, real-world psychiatric contexts.

### Limitations

This study has several limitations. First, although we used multicenter real-world psychiatric EHRs, differences in documentation style—including variability in narrative structure, terminology, completeness of information, and implicit diagnostic cues—may have influenced model performance. These inconsistencies can alter the prominence, clarity, and temporal linkage of symptom descriptions, potentially affecting diagnostic outputs. Broader validation in institutions with diverse linguistic and cultural backgrounds is needed to assess cross-system and cross-national generalizability more fully [31].

Second, this study adopted a single unified prompting strategy to ensure consistent comparability across models. However, previous research has shown that more advanced prompting techniques can improve diagnostic reasoning. For example, Savage et al [32] reported that diagnostic reasoning prompts enhance LLMs’ ability to emulate stepwise clinical reasoning compared with standard prompts. In our setting, a small exploratory pilot study using chain-of-thought prompts on 200 randomly selected cases suggested a modest improvement in exact-match accuracy (about 2-3 percentage points), particularly for mood and anxiety disorder categories. Although this was not a full comparative experiment, the results suggest that the prompting strategy may influence diagnostic performance. Future studies should systematically evaluate zero-shot, few-shot, and chain-of-thought prompt approaches across diagnostic categories and age groups.

Third, the study used a single-label classification framework based on primary discharge diagnoses. While this reflects routine clinical practice, it does not capture psychiatric comorbidities, which are common and clinically important. Multilabel diagnostic frameworks may be necessary to better reflect the real-world complexity of diagnosis.

Fourth, our analysis relied on static EHR text and did not incorporate longitudinal or multimodal clinical information. Real-world psychiatric care involves dynamic symptom progression and treatment trajectories, which cannot be fully captured in static narratives. Integrating time series EHR data, imaging, or laboratory results may increase the diagnostic robustness of LLMs [33].

Fifth, the dataset did not include key socioeconomic variables—such as education level, employment or income status, or urban versus rural residence—which limited our ability to examine potential algorithmic bias or fairness across different social strata. These factors can shape health care access, diagnostic pathways, and linguistic characteristics of clinical documentation, and their absence prevented a systematic assessment of whether model performance varied across sociodemographic groups. Future studies incorporating richer socioeconomic status–related demographic information will be essential for evaluating equity in LLM-assisted diagnostic workflows.

Sixth, although admission diagnoses were removed from model inputs, sections, such as the history of present illness, may still include implicit diagnostic tendencies embedded within physicians’ narrative styles. To address this concern, we manually reviewed a subset of correctly and incorrectly classified cases to determine whether the model relied on “giveaway” phrases or diagnosis-specific linguistic cues. We did not identify consistent or systematic patterns—such as psychopharmacological keywords, standardized phrasing, or clinician-specific formulations—that uniquely pointed to a specific diagnosis. When such terms occurred (eg, references to antidepressants or antipsychotics), they were typically nonspecific and appeared across multiple diagnostic categories.

However, certain unmasked psychotropic medications—particularly lithium and clozapine, which function as strong diagnostic proxies—may have allowed the models to infer diagnoses through medication extraction rather than full clinical reasoning, potentially inflating accuracy in specific categories. These observations suggest that while soft leakage cannot be entirely eliminated in narrative EHRs, its impact on model performance in this dataset was likely limited. Future studies should consider masking medication names or evaluating model behavior under medication-removed conditions to better isolate true diagnostic reasoning.

In addition, this study has privacy-related limitations. Although all EHR narratives were deidentified before transmission to external LLM servers, narrative clinical text carries inherent challenges for complete anonymization. Despite multiple safeguards, the possibility of residual identifiers cannot be entirely eliminated, and future research should prioritize on-premise or institution-governed LLM deployment to avoid external data transmission fully. Moreover, because the manual deidentification audit was conducted on a sampled subset of 500 records rather than the full dataset, a small risk of undetected residual identifiers cannot be completely excluded.

Furthermore, the study did not include a human benchmark for comparison, such as the diagnostic accuracy of junior clinicians, admitting psychiatrists, or structured admission diagnoses. Without this comparative context, it is difficult to ascertain whether the observed strict accuracy of 71.7% represents a clinically meaningful performance threshold. Future research should incorporate human-model comparison frameworks or prospective reader studies to more clearly establish the clinical utility of LLM-assisted diagnostic workflows.

Finally, although multicenter data improved external validity, further validation through prospective observational designs or randomized controlled trials is necessary to robustly establish the real-world clinical utility of LLMs in psychiatric diagnostic workflows [31].

### Future Directions

Future research should expand the diversity of data sources to evaluate the generalizability of LLMs across different health care systems, linguistic environments, and cultural contexts. The incorporation of longitudinal EHR data, time series symptom trajectories, and multimodal clinical information—such as laboratory tests and imaging—may further enhance the ability of LLMs to capture dynamic clinical changes and improve diagnostic robustness.

Furthermore, more sophisticated strategies warrant systematic investigation. Comparative evaluations of zero-shot, few-shot, and chain-of-thought prompts across different psychiatric categories and age groups may help optimize the integration of LLMs into clinical workflows. Future studies should also explore multilabel diagnostic frameworks to capture psychiatric comorbidities better and reflect the complexity of real-world clinical presentations.

Finally, prospective validation—particularly through observational studies or randomized controlled trials—will be essential for establishing the real-world clinical utility, reliability, and safety of LLM-assisted diagnostic tools in psychiatric practice.

### Conclusions

This multicenter real-world study systematically evaluated the diagnostic performance of the GPT-4.0, GPT-3.5, and GLM-4-Plus across all *ICD-10* psychiatric categories using Chinese psychiatric EHRs. GPT-4.0 demonstrated the strongest and most stable diagnostic performance in this study across age groups and diagnostic categories and showed clear advantages in interpreting complex psychiatric narratives. All models performed less reliably in low-prevalence categories, and diagnostic accuracy varied substantially across age groups, with lower accuracy observed in adolescents and young adults.

These findings confirm the feasibility of applying LLMs to real-world psychiatric EHRs and highlight the potential value of GPT-4.0 as a diagnostic support tool. However, the diagnostic outputs of LLMs remain constrained by the quality of documentation, symptom clarity, and the limitations of the models. LLMs should therefore be viewed as complementary tools that can assist clinicians but cannot replace clinical expertise, individualized assessment, or professional decision-making in psychiatric practice.

## Appendix

Multimedia Appendix 1 Instrument and additional tables.

## Abbreviations

EHR: electronic health record
FP: false positive
FN: false negative
ICD-10: International Statistical Classification of Diseases, Tenth Revision
LLM: large language model
OR: odds ratio
TP: true positive

## Data Availability

The datasets generated and analyzed during this study are not publicly available due to patient privacy, but are available from the corresponding author on reasonable request.
