# Supplementary material for: Large Language Models for Psychiatric Diagnosis Based on Multicenter Real-World Clinical Records: Comparative Study
Source: JMIR Med Inform. 2026 Jan 13;14:e77699. doi: 10.2196/77699 (PMC12848494; doi:10.2196/77699)
Supplement: Multimedia Appendix 1 [file medinform_v14i1e77699_app1.docx]

Table S1. Tests of Normality and Homogeneity of Variance.

| Model | Shapiro–W Statistic | Shapiro–W *P* value | Normality |
| --- | --- | --- | --- |
| GPT-3.5 | 0.628 | <.001 | Not normal |
| GPT-4.0 | 0.603 | <.001 | Not normal |
| GLM-4-Plus | 0.626 | <.001 | Not normal |

Levene’s Test for Homogeneity of Variance:

W = 18.30, *P* < .001 → Variances are not equal across groups.

Normality was assessed using the Shapiro–Wilk test, which showed significant deviations from normality in all three model groups (all *P* < .001). Homogeneity of variance was evaluated using Levene’s test, which also indicated significant differences across groups (W = 18.30, *P* < .001). These findings justified the use of non-parametric rank-sum tests for inter-group comparisons.

Table S2. Diagnostic Accuracy of GPT-4.0, GPT-3.5, and GLM-4-Plus Across Six Centres.

|  |  | GPT-4.0 | | | GPT-3.5 | | | GLM-4-Plus | | |
| --- | --- | --- | --- | --- | --- | --- | --- | --- | --- | --- |
| Center | Cases | Correct | Partially correct | Incorrect | Correct | Partially correct | Incorrect | Correct | Partially correct | Incorrect |
| A | 1654 | 1187 (71.8%) | 270 (16.3%) | 197 (11.9%) | 1138 (68.8%) | 266 (16.1%) | 250 (15.1%) | 1146 (69.3%) | 295 (17.8%) | 213 (12.9%) |
| B | 1653 | 1186 (71.7%) | 269 (16.3%) | 198 (12.0%) | 1136 (68.7%) | 266 (16.1%) | 251 (15.2%) | 1145 (69.3%) | 294 (17.8%) | 214 (12.9%) |
| C | 1654 | 1186 (71.7%) | 270 (16.3%) | 198 (12.0%) | 1137 (68.7%) | 267 (16.1%) | 250 (15.1%) | 1146 (69.3%) | 295 (17.8%) | 213 (12.9%) |
| D | 1653 | 1187 (71.8%) | 269 (16.3%) | 197 (11.9%) | 1138 (68.8%) | 265 (16.0%) | 250 (15.1%) | 1147 (69.4%) | 294 (17.8%) | 212 (12.8%) |
| E | 1655 | 1186 (71.7%) | 269 (16.3%) | 200 (12.1%) | 1137 (68.7%) | 266 (16.1%) | 252 (15.2%) | 1145 (69.2%) | 295 (17.8%) | 215 (13.0%) |
| F | 1654 | 1184 (71.6%) | 270 (16.3%) | 200 (12.1%) | 1137 (68.7%) | 264 (16.0%) | 253 (15.3%) | 1144 (69.2%) | 292 (17.7%) | 218 (13.2%) |
| Total | 9923 | 7116 (71.7%) | 1617 (16.3%) | 1190 (12.0%) | 6823 (68.8%) | 1594 (16.1%) | 1506 (15.2%) | 6873 (69.3%) | 1765 (17.8%) | 1285 (12.9%) |

Supplementary Note:

Table S2 presents the centre-level diagnostic performance of the three LLMs. Diagnostic accuracy varied slightly across the six centres but showed no statistically significant differences for any of the models (GPT-4.0: *P* = .519; GPT-3.5: *P* = .472; GLM-4-Plus: *P* = .611, Kruskal–Wallis tests). These results indicate that model performance remained stable and consistent across institutions, supporting the robustness of the multicentre real-world dataset.

Table S3. Ordinal logistic regression results of model performance based on the 0–2 scoring system.

| Comparison | Odds Ratio (OR) | 95% CI (Lower–Upper) | *P* |
| --- | --- | --- | --- |
| GPT-4.0 vs GPT-3.5 | 1.18 | 1.11–1.25 | <.001 |
| GLM-4-Plus vs GPT-3.5 | 0.95 | 0.90–1.01 | 0.092 |

Note: Ordinal logistic regression was performed using the 0–2 scoring system (0 = incorrect, 1 = partially correct, 2 = correct) as the outcome variable. GPT-3.5 was used as the reference category. Odds ratios represent the relative likelihood of obtaining a higher diagnostic score. GPT-4.0 demonstrated significantly better performance than GPT-3.5 (OR = 1.18), whereas GLM-4-Plus showed no statistically significant difference from GPT-3.5 (OR = 0.95, *P* = .092).
